# Cutaneous leishmaniasis in north-central Ethiopia: trend, clinical forms, geographic distribution, and determinants

**DOI:** 10.1186/s41182-020-00231-w

**Published:** 2020-06-03

**Authors:** Belayneh Eshetu, Hassen Mamo

**Affiliations:** 1 Department of Zoological Sciences, College of Natural and Computational Sciences, Addis Ababa University, PO Box 1176, Addis Ababa, Ethiopia; 2 Department of Microbial, Cellular and Molecular Biology, College of Natural and Computational Sciences, Addis Ababa University, PO Box 1176, Addis Ababa, Ethiopia

**Keywords:** Cutaneous leishmaniasis, Prevalence, Determinants, Adjusted odds ratio, Dermatology

## Abstract

**Background:**

Cutaneous leishmaniasis (CL), being among the neglected tropical diseases, catches little attention despite its considerable influence. This study aimed at estimating the prevalence and associated factors of CL in Boru Meda Hospital, Dessie town, north-central Ethiopia.

**Methods:**

Medical records of patients who attended the Dermatology Department of the Hospital in 2012–May 2018 were assessed. In addition, dermatological patients who were visiting the hospital during the data collection period (November 2017–May 2018) were interviewed to capture socio-demographic, environmental variables, and related factors. The source population was individuals who visited the hospital for skin problems in the stated years and CL positives were the targets. The association between CL and its determinants was tested by logistic regression.

**Results:**

CL prevalence was 1.5% showing increasing trend with the year of examination. Localized, diffused, and mucosal CL was evident across the years. Dessie town had the highest prevalence, 291 (32.8%) patients out of 888 cases. The number of examined (29,701) and positives (543, 1.8%) for males was comparable with females, 28,459 and 345 (1.2%), respectively, increasing with age but without significant difference. Dessie town residence (adjusted odds ratio (AOR) 12.2, 95% confidence interval (CI) 2.2–18.6, *p* = 0.01), no bed net (AOR 9.9, 95% CI 2.7–16.7, *p* < 0.01), nearby irrigation (AOR 8.1, 95% CI 1.9–12.4, *p* < 0.01), and travel to CL endemic areas (AOR 13.9, 95% CI 4.4–14.3, *p* < 0.01) were significantly associated with CL.

**Conclusion:**

CL is a growing health problem in Dessie and its surroundings. Known risk factors prevail. Comprehensive parasitological, entomological, and social studies are warranted to better manage the disease.

## Introduction

Leishmaniasis is a collective name of diseases caused by different species of the intracellular protozoa of the genus *Leishmania* [1] and transmitted by the bite of female phlebotomine sandflies of the genera *Phlebotomus* or *Lutzomyia* [2]. Human leishmaniasis is manifested in distinct clinical forms ranging from mild self-healing cutaneous lesions or nodules termed cutaneous leishmaniasis (CL) to a fatal systemic illness called visceral leishmaniasis (VL) or more commonly kala-azar. In between, there is mucocutaneous leishmaniasis (MCL) or “espundia” and post-kala-azar dermal leishmaniasis—PKADL [3]. MCL, which is primarily caused by *Leishmania braziliensis braziliensis* and most commonly existing in Bolivia, Brazil, and Peru, affects the mucosa membrane and if untreated may be fatal invariably [4]. VL caused by *L. donovani* complex is a febrile malaria-resembling illness characterized by swelling of the lymph nodes, the liver, and spleen (hepatosplenomegaly) and anemia, emaciation, and eventually death in the absence of intervention [5]. VL is a widely distributed tropical and subtropical disease occurring in almost all continents with the possible exception of Australia although the highest burden is borne in Bangladesh, Brazil, India, Nepal, and Sudan [6].

However, CL is by far the most common form of leishmaniasis mainly caused by *L. major*, *L. tropica*, and *L. aethiopica* in Europe (the Mediterranean basin), Asia (the Middle East and Central Asia) and Africa, and *L. mexicana* in the Americas. In Asia, CL is predominantly found in Afghanistan, Iran, Saudi Arabia, and Syria; in Africa, in Algeria, and in South America Brazil, Colombia and Peru take the biggest share. About 95% of CL cases with over two thirds of new CL cases occur in these countries [7]. Overall, CL is currently endemic in 88 countries globally (20 countries in South and Central America and 68 in Europe, Africa, the Middle East, Central Asia, and the Indian subcontinent). An estimated 700,000 to 1,000,000 new cases and some 26,000 to 65,000 deaths occur annually, but only 19–37% is actually reported to health authorities although there are an estimated 12 million cases worldwide [7].

Although leishmaniasis is considered the sixth most significant disease in the tropics/subtropics by the World Health Organization (WHO), CL remains among the neglected tropical diseases (NTDs) [8]. As CL leaves a disfiguring scar on the patient’s body, it is a cause of lifelong stigma and discrimination leading to significant psychosocial problems [9, 10]. Currently, the incidence of CL is growing and projected to be more in the future for factors related to poverty and its various manifestations [11], climatic/environmental changes [12], drug-resistance [13], exoduses because of conflicts [14, 15], and immunodeficiency particularly due to AIDS [16].

Since its first description by the Italian epidemiologist Martoglio in 1913 [17], several authors in the late 1960s and early 70s (Balzer et al. 1960 (in [18–20])) reported the occurrence of CL in a number of localities in Ethiopia and described its epidemiology. Subsequent surveys further established the endemicity of CL in most highlands of Ethiopia with preponderance in areas 1400–2700 m above sea level. To mention a few, wide CL foci occur in different parts of Tigray [21–24] and Gondar [25] in the north and northwest, Dembidolo (Wollega) in the west [26], Silti [27], Sidamo [28] and Ocholo (Gamo Gofa) in the south [29–31], and Addis Ababa and its surroundings in the center [32, 33]. CL has different vernacular names in different localities of Ethiopia like *bolbo* in Ocholo, *finchottu* in Central Shoa, *shahegne* in North Shoa, *kunchir* in Gojam, Gondar and parts of Wollo, *giziwa* in Tigray, *chewie* in Sodo, and *simbirahalkm* in Wollega as reviewed in Seife et al. 2018 [34]. The majority of CL cases in Ethiopia are due to *L. aethiopica* [35] though there were some cases by *L. major* and *L. tropica* [36].

Several people from Dessie town, north-central Ethiopia, and the surrounding areas visit Boru Meda Hospital (BMH) for skin diseases of multiple etiologies including CL. Furthermore, people with cured scars or active CL lesions are visible in schools, on the street, markets, and other places in the town. However, there is no recent information on the magnitude of CL in and around Dessie, except an attempt on the treatment efficacy of anti-leishmaniasis agents on a limited number of patients (*n* = 97) in BMH [34]. This study was aimed at assessing the prevalence of CL among the patients attending BMH and the associated risk factors. Medical reports, combined with future field works and molecular analyses as well as entomological surveys will help identify the status of local transmission of CL and its spatial and temporal distribution. Such an investigation will provide better insights into the problem and contribute towards designing sustainable CL management strategies in Dessie and its environs.

## Methods

### The study area

The study area is Dessie town in north-central Ethiopia some 388 km from Addis Ababa with latitude and longitude of 11° 8′ N 39° 38′ E and 2470–2550 m above sea level. Dessie was established by King Michael in 1893 currently covering an area of 16000 ha. It is divided into 10 sub-cities and 6 rural *kebeles* (the lowest administrative unit). The study was conducted in BMH. According to the information obtained from the Hospital, BMH was established in 1955 by the Sudan Interior Mission (SIM). During its establishment, it was intended that the hospital would provide ophthalmological and dermatological services. After serving for about 40 years as per its initial objective, the hospital was upgraded to deliver comprehensive medical services now. Around 2.5 million people living in South and North Wollo Zones, Oromia Special Zone, North Shewa, South Tigray, and parts of Afar Region are served by the hospital. The hospital remained the only well-recognized center for skin disease management in the eastern part of the Amhara Region and beyond.

### Study design, population, and data collection

Patients who presented to BMH Dermatology Department between October 2017 and May 2018 were recruited and interviewed to gather important socio-demographic and other related information, and their CL status was determined clinically and/or parasitologically (Giemsa stains of skin slit smear or fine-needle aspiration cytology (FNAC)/skin biopsy) as well as culture. The questionnaire was adopted from the literature and used with slight modification. The original English version of the questionnaire was translated first into Amharic and back into English to assure consistency. Then, it was administered to patients who came to the hospital for CL and other skin disease treatment. The collected data were checked for completeness, accuracy, clarity, and consistency. Additionally, the medical records of individuals who visited the hospital in the preceding years (January 2012–September 2017) for the same problem were considered and CL-positive cases (clinical/parasitological/culture) identified and socio-demographic data extracted.

### Data analysis

While the dependent variable was CL positivity, the independent variables constituted socio-demographics such as age, sex, knowledge, residence area, and related household and environmental factors. The differences between age and gender categories as well as residence, which were the only available variables for the retrospective data, with regard to CL have been tested by the chi-square test. Univariable and multivariable logistic regression tests at 95% confidence intervals (CI) were used to assess the association between the dependent and independent variables for the interviewed patients only. Statistical significance was at *p* < 0.05. The statistical analysis was carried out using the Statistical Package for Social Sciences (SPSS IBM Statistics) version 20.

## Results

### CL cases by age and gender

In the past six and half year (January 2012–May 2018), totally 58,163 people were examined in the Dermatology Department of BMH. Of these, 29,704 were males and 28,459 females (male to female ratio nearly 1:1). The total prevalence of CL was 1.5% with 543 (1.8%) males and 345 (1.2%) females (Table 1). The rate of CL increased from 0.9% in 2012 to 3.5% in 2018. Overall as well as throughout, slightly more males were examined for skin problems except for the slightly higher number of females in 2016 and 2017. Among the CL positives (*n* = 888), 543(61.1%) were males and 345(38.9%) females. There was a slightly higher prevalence of CL positivity in males than in females in all the years considered although the difference was not statistically significant.

**Table 1:** Prevalence of CL among patients who visited the BMH Dermatology Department from 2012 to 2018

**Year**	**Examined**			**CL positive**		
	Male, *n* (%)	Female, *n* (%)	Total	Male, *n* (%)	Female, *n* (%)	Total, *n* (%)
2012	2327 (53.6)	2016 (46.4)	4343	26 (1.1)	15 (0.7)	41 (0.9)
2013	3363 (51.8)	3129 (48.2)	6492	27 (0.8)	14 (0.5)	41 (0.6)
2014	3181 (54.6)	2644 (45.4)	5825	34 (1.1)	13 (0.5)	47 (0.8)
2015	4779 (53.8)	4102 (46.2)	8881	29 (0.6)	23 (0.6)	52 (0.6)
2016	4664 (49.9)	4683 (50.1)	9347	38 (0.8)	30 (0.6)	68 (0.7)
2017	7507 (48.4)	8014 (51.6)	15,521	221 (2.9)	149 (1.9)	370 (2.4)
2018*	3883 (50.1)	3871 (49.9)	7,754	168 (4.3)	101 (2.6)	269 (3.5)
Total	29,704 (51.1)	28,459 (48.9)	58,163	543 (1.8)	345 (1.2)	888 (1.5)

*BMH* Boru Meda Hospital, *CL* cutaneous leishmaniasis, *n* number, *%* per cent

*2018 data was only up to May

The proportion of CL-positive individuals was different in different age groups for both genders. In general, there was an increasing trend of CL cases with age and in both genders; those over 14 years were the most affected. While 3.4%, 11.6%, and 46.2% of under-5, 5–14 and > 14-year-old males were CL-positive, the corresponding proportions of females were 2.7%, 9.5%, and 26.7% (Table 2).

**Table 2:** CL in different age and sex groups among patients who visited the BMH Dermatology Department between 2012 and May 2018 (*N* = 888)

Age (year)	Sex	Year							
		2012 *n* (%)	2013 *n* (%)	2014 *n* (%)	2015 *n* (%)	2016 *n* (%)	2017 *n* (%)	2018* n (%)	Total *n* (%)
< 5	Male	1 (3.3)	1 (1.3)	1 (1.3)	4 (13.3)	0 (0.0)	16 (53.3)	7 (23.3)	30 (3.4)
	Female	0 (0.0)	1 (4.2)	0 (0.0)	1 (4.2)	3 (12.5)	12 (50.0)	7 (27.2)	24 (2.7)
**Total**		**1 (1.9)**	**2** (**3.7)**	**1 (4.2)**	**5 (9.3)**	**3 (5.7)**	**28 (51.9)**	**14 (25.9)**	**54 (6.1)**
5–14	Male	7 (6.8)	6 (5.8)	6 (5.8)	4 (3.9)	7 (6.8)	47 (45.6)	26 (25.2)	103 (11.6)
	Female	2 (2.4)	2 (2.4)	4 (4.8)	5 (5.9)	7 (8.3)	45 (53.6)	19 (22.6)	84 (9.5)
**Total**		**9 (4.8)**	**8 (4.3)**	**10 (5.4)**	**9 (4.8)**	**14 (7.5)**	**92 (49.2)**	**45 (24.1)**	**187 (21.1)**
> 14	Male	18 (4.4)	20 (10.7)	27 (6.6)	21 (5.1)	31 (7.6)	159 (38.8)	134 (32.7)	410 (46.2)
	Female	13 (5.5)	11 (4.6)	9 (3.8)	17 (7.2)	20 (8.4)	92 (38.8)	75 (31.7)	237 (26.7)
**Total**		**31 (4.8)**	**31 (4.8)**	**36 (5.6)**	**38 (5.9)**	**51 (7.9)**	**251 (38.8)**	**209 (33.3)**	**647 (72.9)**
Overall	Male	26 (4.8)	27 (4.9)	34 (6.3)	29 (5.3)	38 (6.9)	221 (40.7)	168 (30.9)	543 (61.2)
	Female	15 (4.4)	14 (4.1)	13 (3.8)	23 (6.7)	30 (8.7)	149 (43.2)	101 (29.3)	345 (38.9)
**Total**		**41 (4.6)**	**41 (4.6)**	**47 (5.3)**	**52 (5.9)**	**68 (7.7)**	**370 (41.7)**	**269 (30.3)**	**888 (100)**

*BMH* Boru Meda Hospital, *CL* cutaneous leishmaniasis, *n* number, *%* percent

*2018 data was only up to May

### Patient place of residence

**Fig. 1:**
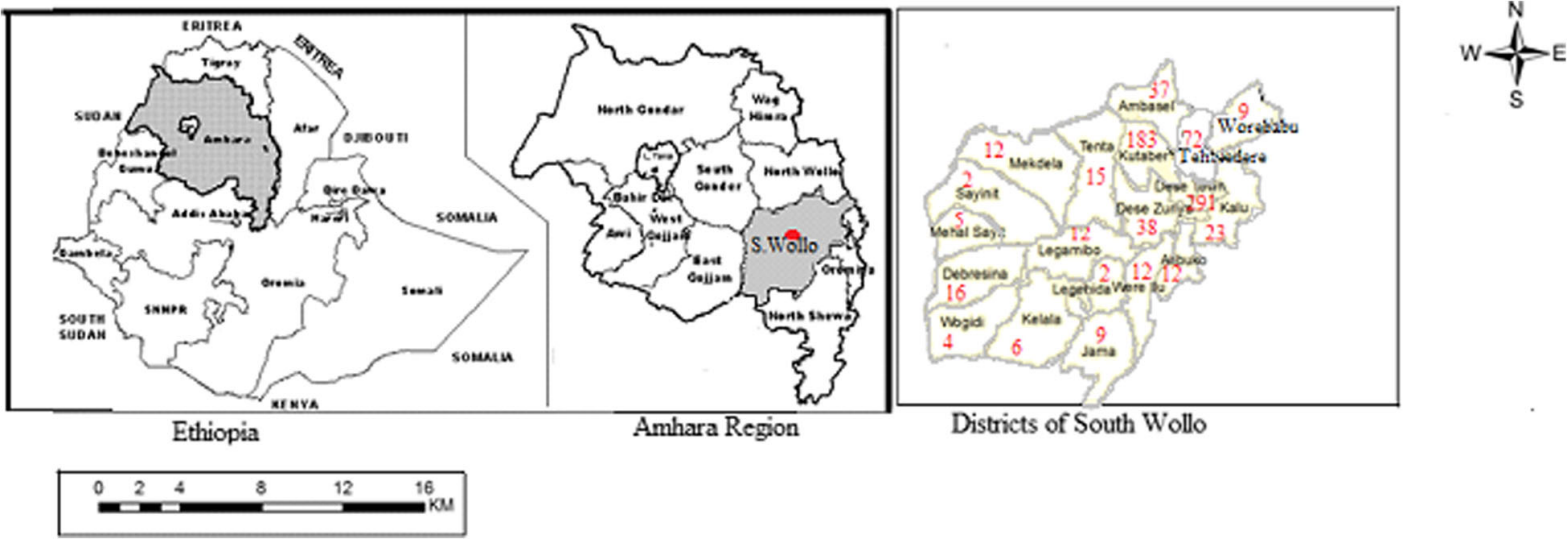
Map of Ethiopia, Amhara Region, and South Wollo Zone Districts where from cutaneous leishmaniasis (CL) patients of Boru Meda Hospital originated. The figure inside each district indicates the number of CL cases**. Since Dessie town and Kutaber are closer to the Hospital, more people are expected to be examined and correspondingly more are CL-positive. So, unless the number of people examined is known it may be misleading to conclude that the highest number and proportion of CL were from these districts**

The CL cases in the Hospital were from about thirty-three districts including Dessie town. Nineteen of these are South Wollo Zone districts; all the districts in the Zone had CL cases. The remaining thirteen are from North Wollo (9), North Shoa (2), and Oromia Special Zone (2) which share borders with South Wollo. One district having three CL cases belongs to Tigray Region and is located at North Wollo boundary. Quantitatively, 789 (89.0%) of the CL cases were from South Wollo, 74 (8.0%) from North Wollo, 13 (2.0%) North Shewa, and 9 (1.0%) from Oromia. The proportion of CL cases from Dessie town was the highest (291 (33.0%) followed by Kutaber (183 (21.0%) and Tehulederie (72 (8.0%) that are within the immediate vicinity (Fig. 1).

### Repeated and new CL cases, type, and location of lesion

Among patients of CL who were examined in the Hospital, 521(58.7%) were new cases – 318 (58.6%) males and 203 (58.8%) females. The rest 367(41.3%) were previously treated cases. That is, males and females who took treatment more than once in the Hospital for CL were respectively 225 (61.3%) and 142 (38.7%). The number and proportion of newly (once) and repeatedly treated (relapse) individuals in the hospital had shown variation with the year of examination. Specifically, 68.3%, 75.6%, 74.1%, 71.5%, 80.9%, 54.9%, and 49.1% attended treatment once and 31.7%, 24.4%, 25.5%, 28.9%, 19.1%, 45.1%, and 50.9% treated more than once during the study period from 2012 to May 2018, respectively. The data showed that more repeat-cases were observed in 2017 and 2018 than the rest of the years (Table 3).

**Table 3:** New and repeat CL cases among patients who were treated at the BMH Dermatology Department from 2012 to May 2018

CL cases	Year of examination																
	2012		2013		2014		2015		2016		2017		2018		Total		Overall
	M	F	M	F	M	F	M	F	M	F	M	F	M	F	M	F	
**Repeat**	8	5	7	3	9	3	10	5	7	6	90	77	94	43	225	142	367
**New**	18	10	20	11	25	10	19	18	31	24	131	72	74	58	318	203	521
**Total**	**26**	**15**	**27**	**14**	**34**	**13**	**29**	**23**	**38**	**30**	**221**	**149**	**168**	**101**	**543**	**345**	**888**

*CL* cutaneous leishmaniasis, *F* female, *M* male

Out of the 888 patients, 792 (89.2%) were localized cutaneous leishmaniasis (LCL), 35 (3.9%) diffuse cutaneous leishmaniasis (DCL) and 61(6.9%) were MCL cases. The data showed that the majority of the cases were localized, particularly at the nose and cheek. The duration of lesions, treatment, and treatment outcomes was not available within patient medical records. Similarly, data for the four different diagnostics (clinical, skin slit smear, FNAC, and culture) that are reported practiced in the hospital were not available.

### Questionnaire data

The questionnaire data showed that out of 354 respondents, 216 (61.0%) were males and 138 (38.9%) females. Twenty-four (6.8%) of the respondents were under-5 children, 52 (14.7%) 5–14 years and the rest 278 (78.5%) over 14 years. Most (141) of the respondents were secondary school students, 89 at primary school, 72 joined college and university education, and the rest 52 had no formal schooling. Hundred seventy-nine of the respondents (50.6%) were CL-positive (111 males and 68 females). Most (76 or 42.5%) of the CL-positive individuals were secondary school students, 34 (19.0%) without schooling, 45 (25.1%) primary school attending, and the rest 24 (13.4%) had tertiary education.

Out of 197 (55.7%) students, 54 (15.3%) farmers, 52 (14.7%) civil servants, and 51 (14.4%) merchants; CL-positive individuals were 103 (55.5%), 37 (20.7%), 17 (9.5%), and 22 (12.3%), respectively. From student respondents 52.3% individuals, among farmer participants 68.5%, from merchants 43.1%, and from the governmentally employed respondents 32.7% were CL-positive patients. The data showed that CL cases were greatest in farmers and least among civil servants.

Only 83 respondents knew what CL meant and 34 had knowledge about its transmission. Among the respondents, 116 (32.8%) responded their living houses were built from muddy and grassy materials and 183 (51.7%) were living in houses with cracked walls and only 53(14.9%) lived in houses with moist floors. Concerning sleeping outside under a tree, only 36 (10.2%) agreed that they sleep outside under trees. However, 318 (89.8%) people sleep inside. Only 27 (7.6%) of the respondents responded that they use bed nets. There were dogs and other animals like cattle in the houses of 248 (70.1%) of the respondents. Ninety-three (26.3%) were living near irrigation areas, 54 (15.3%) near open sewage areas, and 54 (15.3%) had traveled to CL endemic areas.

Among the variables tested in the univariate analysis most were significantly associated with CL positivity except gender, age, sleeping under the tree, and being a merchant or living outside Dessie town or Kutaber (Table 4).

**Table 4:** Univariable logistic regression analysis of potential determinants in relation to CL positivity at BMH, Dessie town, Northeast Ethiopia, November 2017–May 2018 (*N* = 354)

Variable	Alternatives	***N***	Pos (%)	COR	95% CI	***P*** value
Gender	Male	216	111 (51.4)	0.9	0.6-1.4	0.69
	Female	138	68 (49.3)	1.0	–	–
Age (year)	< 5	24	3 (12.5)	1.0	–	–
	5–14	52	16 (30.8)	1.2	0.6–2.1	0.62
	> 14	278	160 (57.6)	1.5	0.9–2.6	0.07
Residence	Dessie town	137	80 (58.4)	3.9	1.2–12.7	0.02
	Kutaber	79	46 (58.2)	3.8	1.1–13.1	0.03
	Tehulederie	34	17 (50.0)	2.8	0.7–10.3	0.13
	Other South Wollo areas	17	9 (52.9)	3.1	0.7–13.7	0.13
	Delanta	23	9 (39.1)	1.8	0.4–7.2	0.43
	Ambasel	18	7 (38.9)	1.8	0.4–7.7	0.46
	Dessie Zuria	19	5 (26.3)	0.9	0.2–4.6	0.98
	South Wollo border areas	15	4 (26.7)	0.6	0.1–3.7	0.53
	Kalu	12	2 (16.7)	1.0	–	–
Education	No formal schooling	52	34 (65.4)	3.8	1.8–8.1	0.00
	Primary school	89	45 (50.6)	2.0	1.1–3.9	0.02
	Secondary school	141	76 (53.9)	2.3	1.3–4.2	0.00
	Tertiary education	72	24 (33.3)	1.0	–	–
Job	Civil servant	52	17 (32.7)	1.0	–	–
	Farmer	54	37 (68.5)	4.5	1.9–10.1	< 0.01
	Student	197	103 (52.3)	2.3	1.2–4.3	0.01
	Merchant	51	22 (43.1)	1.6	0.7–3.5	0.27
Know what CL is	Yes	83	27 (32.5)	1.0	–	–
	No	271	152 (56.1)	2.6	1.6–4.4	< 0.01
Know CL transmission	Yes	34	5 (14.7)	1.0	–	
	No	320	174 (54.4)	6.9	2.6–18.3	< 0.01
Know factors related to CL	Yes	32	5 (15.6)	1.0	–	
	No	322	174 (54.0)	6.3	2.4–16.9	< 0.01
Muddy-grassy houses	Yes	116	85 (73.3)	4.2	2.6–6.8	< 0.01
	No	238	94 (39.5)	1.0	–	
Wall cracks	Yes	183	122 (66.7)	3.9	2.5–6.0	< 0.01
	No	171	57 (33.3)	1.0	–	
Moist floor	Yes	53	36 (67.9)	2.8	1.3–4.3	0.00
	No	301	143 (47.5)	1.0	–	
On-floor sleeping	Yes	47	42 (89.4)	10.4	4.0–7.0	< 0.01
	No	307	137 (44.6)	1.0	–	–
Bed net use	Yes	27	4 (14.8)	1.0	–	–
	No	327	175 (53.5)	6.6	2.2–9.6	0.01
Sleeping under tree	Yes	36	23 (63.9)	1.8	0.9–3.8	0.09
	No	318	156 (49.1)	1.0	–	–
Open sewage nearby house	Yes	54	37 (67.5)	2.4	1.3–4.5	0.00
	No	300	142 (47.3)	1.0	–	–
Irrigation nearby house	Yes	93	84 (90.3)	16.3	7.8–13.9	< 0.01
	No	261	95 (36.4)	1.0	–	–
Traveled to CL endemic areas	Yes	54	46 (85.2)	7.2	3.3–15.8	< 0.01
	No	300	133 (44.3)	1.0	–	–
Own dogs and other animals	Yes	248	137 (55.4)	1.9	1.2–2.9	0.00
	No	106	42 (39.6)	1.0	–	–

Note: *n* number, *Pos* positive, *COR* crude odds ratio, *CI* confidence interval, significant at *p* < 0.05

In the multivariable model, Dessie town residence, lack of CL knowledge, living in muddy and grassy houses or cracked-wall houses, lack of bed net, irrigation nearby, possession of dogs or other animals, and travel history to CL endemic areas were significantly associated with CL occurrence (Table 5).

**Table 5:** Multivariable logistic regression analysis result of risk factors for CL positivity at BMH, Dessie town, north-central Ethiopia, November 2017–May 2018 (*N* = 354)

Variable	Categories	***n***	Pos, ***n*** (%)	AOR	95% CI	***P*** value
Residence district	Dessie town	137	80 (58.4)	12.2	2.2–18.6	0.00*
	Kutaber	79	46 (58.2)	11.6	0.9–16.2	0.06
	Other South Wollo areas	17	9 (52.9)	11.6	0.8–17.4	0.07
	Tehulederie	34	17 (50.0)	10.4	0.5–19.8	0.13
	Delanta	23	9 (39.1)	10.2	0.5–18.5	0.13
	Ambasel	18	7 (38.9)	6.9	0.5–10.3	0.15
	South Wollo border areas	15	4 (26.7)	5.14	0.3–7.9	0.23
	Dessie Zuria	19	5 (26.3)	2.2	0.1–6.5	0.62
	Kalu	12	2 (16.7)	1.0	–	–
Education	No formal schooling	52	34 (65.4)	4.4	0.8–5.1	0.093
	Primary school	89	45 (50.6)	0.9	0.9–3.5	0.8
	Secondary school	141	76 (53.9)	1.2	0.2–3.5	0.8
	Tertiary	72	24 (33.3)	1.0	–	–
Job	Civil servant	52	17 (32.7)	1.0	–	–
	Merchant	51	22 (43.1)	2.3	0.4–15.6	0.33
	Student	197	103 (52.3)	2.5	0.4–14.7	0.28
	Farmer	54	37 (68.5)	3.1	0.5–11.4	0.31
Know what CL is	Yes	83	27 (32.5)	1.0	–	–
	No	271	152 (56.1)	3.9	1.3–11.4	0.01*
Know CL transmission	Yes	34	5 (14.7)	1.0	–	–
	No	320	174 (54.9)	3.4	0.9–13.7	0.08
Know factors related to CL	Yes	32	5 (15.6)	1.0	–	–
	No	322	174 (54.0)	2.7	0.6–12.3	0.21
Muddy-grassy house	Yes	116	85 (73.3)	4.2	1.7–10.5	0.00*
	No	238	94 (39.5)	1.0	–	–
House wall cracks	Yes	183	122 (66.7)	3.2	1.4–7.0	0.00*
	No	171	57 (33.3)	1.0	–	
On-floor sleeping	Yes	53	36 (67.9)	1.7	0.7–4.4	0.32
	No	301	143 (47.5)	1.0	–	–
Bed net use	Yes	27	4 (14.8)	1.0	–	–
	No	327	175 (53.5)	9.9	2.7–16.7	< 0.01*
Sleeping under tree	Yes	244	147 (60.3)	5.2	2.2–12.3	< 0.01*
	No	110	32 (29.1)	1.0	–	–
Open sewage nearby house	Yes	54	37 (67.5)	2.5	0.9–6.9	0.06
	No	300	142 (47.3)	1.0	–	–
Irrigation nearby house	Yes	93	84 (90.3)	8.1	1.9–12.4	< 0.01*
	No	261	95 (36.4)	1.0	–	–
Traveled to CL endemic areas	Yes	54	46 (85.2)	13.9	4.4–14.3	<0.01*
	No	300	133 (44.3)	1.000	–	–
Own dogs and other animals	Yes	248	137 (55.2)	2.272	1.0–5.1	0.04*
	No	106	42 (39.6)	1.000	–	–

*n* number, *%* per cent, *Pos* positive, *AOR* adjusted odds ratio, *CI* confidence interval

*Significant at *p* < 0.05

## Discussion

The overall prevalence of CL among patients who visited the BMH Dermatology Department from 2012 to May 2018 was 1.5%. This is much lower than the prevalence reported by similar health facility-based studies in Ethiopia such as Addis Ababa 14.2% [33]. Although the nationwide prevalence of CL is lacking in Ethiopia, there are some community-based cross-sectional surveys showing 2.3–14.0% prevalence [15, 23, 24, 27] from various parts demonstrating remarkable heterogeneity. A meta-analysis and systemic review [37] reported a pooled prevalence of 19.0%. The relatively lower prevalence of CL in the study area might be due to a number of reasons. Previously, some people might follow traditional medication options. Others used to visit the hospital, diagnosed but referred to other hospitals where leishmaniasis treatment service is readily available. However, recently, the hospital has started serving CL patients as any other skin disease cases (personal communication, hospital staff). During the time of the study, several people were attending the hospital for CL treatment from different nearby as well as distant areas. The increased prevalence of CL cases in 2017 and up to May 2018 corroborates this explanation. The CL prevalence increased from 0.9% in 2012 to 3.5% in 2018 to May.

The percentage of CL-positive males was 1.8% which was comparable with females (1.2%) agreeing with a report from different localities of Ethiopia at different times [19, 24, 30], and in some countries like Saudi Arabia in the Middle East [38] where no significant gender disparity was recorded. Further, a study in Argentina from South America [39] reported no significant difference between males and females suggesting equal exposure to infection.

On the other hand, a number of findings indicated that the male gender is a risk factor for CL in different countries (reviewed in ref no. [38, 40], and [41] including Ethiopia [22]). The authors attribute this to variations in the activities of males and females and thus exposure to the sandfly vectors, provided there is no significant difference in gender attendance to health services. Males may have closer contact with the habitat of the sandfly through occupation and leisure activity. Cultural activities that mostly males are faced with outdoor activities including farming, keeping cattle, staying around gorges, and/or farmland for a long period and the presence of endemic sites that mostly males could travel there for work.

Furthermore, the gender difference in CL incidence is attributable to sex hormonal effects or immune responses [42], as it has been noted in some other parasitic diseases [43]. Differences in exposure or access to healthcare per se do not necessarily explain gender-based differences for infection or clinical disease. The role of gender in reference to infectious diseases is demonstrated in an animal model as well [44]. In general, there is a strong correlation between gender and incidence of leishmaniasis and the disease is more frequent among men than women although the current study did not suggest that.

The secondary data collected from the hospital records showed that the total percentage of CL positive individuals was different between age groups. Out of 888 CL-positive individuals, 6.1%, 21.1%, and 72.9% were under-5, 5–14, and ≥ 15, respectively, although the number of examined in each group is unknown. The primary data also revealed an almost similar pattern in relation to age although in the logistic regression age was not identified as a significant risk factor. This might be due to the outdoor activities performed by individuals of this age group in farms and the likes than individuals below this age as they are actively working groups.

Nevertheless, the age-related pattern of CL attack is variable across studies in Ethiopia. In some, the more predominantly affected group is the younger 0–9 [24], in others, the 11–20 [27], > 14 [22], or those 10–19 years old [23]. The data showed that the majority (89.2%) of the CL cases were localized, particularly at the nose and cheek. Diffuse and mucocutaneous accounted only for 3.9% and 6.9%, respectively. It is known that sandfly vectors bite in the face, which is exposed, and cutaneous lesions appear in the site of promastigote inoculation following the bite. In other localities and countries, the face is noticed as the most infected organ by the *Leishmania* although some other studies showed leishmaniasis lesions in the hands and feet as well [45].

The current hospital data indicates that CL cases were observed in 35 different districts including Dessie town. There was a greater variation of CL prevalence amongst the different districts. The data showed that the highest CL prevalence was in Dessie town. The high prevalence of CL in Dessie town and Kutaber district could be due to the higher altitude of 2500 m above sea level together with other factors such as recent urbanization, especially in Dessie. CL is distributed mainly at high and mid altitudes ranging from 1400 to 2700 m above sea level, which is most favorable for the proven vector sandfly species. In a recent mapping study in Ethiopia [46], CL occurrence was significantly associated with an altitudinal range between 810 and 3563 m above mean sea level increasing slope values producing higher CL correlations. The results show the widespread endemicity of CL in Dessie town and its surroundings calling for future field-based epidemiological surveys. Previous reports concerning the endemicity of CL in Kutaber, which is some 10 km from Dessie town [18] and “Dessie area” [19], exist, but this report is the first of its kind for CL from Dessie town proper in recent times. The urbanization process of the town, which is a well-known risk factor for CL, is ongoing [47].

The data showed that the CL prevalence was highest for farmers (68.5%) and least among civil servants. Farming activity could put farmers at greater risk of CL than non-farmers such as government employees. In the farm field and irrigation areas, and possible travel to endemic areas and other seasonal activities farmers could be more exposed to sandfly bites. Students are second in CL prevalence because most students are families of farmers and engaged in similar activities as their parents.

Lack of CL knowledge, living in houses constructed of mud and grass, presence of cracks in house wall, sleeping on the floor, lack of using bed net during sleeping, presence of plants and irrigation areas nearby houses, presence of dogs and other animals in the compound or living houses, and travel history to endemic areas are significantly associated with CL. The responses of the participants on the knowledge-based questionnaire showed that the majority of the respondents were not aware of the meaning, transmission, and associated risk factors of CL, and this lack of knowledge was significantly associated with CL. This result is in agreement with reports and reviews by various authors [48–51].

## Conclusion

In the study area, CL prevalence was progressively increasing within the past six and half year. Moreover, the study demonstrated that CL remained endemic in Dessie town and its surroundings without being well documented in the literature. Common CL risk factors incriminated elsewhere are prevalent in the study area. The study revealed that the community lacked awareness about the meaning, transmission, and associated risk factors of CL. To better control and monitor CL, awareness creation is necessary. The hospital lacked organized documentation system about CL patients and requires improvement particularly in the specific diagnostics ordered for a particular patient, drugs prescribed, and treatment outcomes as well as detailed clinical features, for instance, the duration of the lesions including whether dry, wet, painful, or painless. Comprehensive future studies on the epidemiology and public health burden of the disease in the area are essential.

## Data Availability

All data generated or analyzed during this study are included in this published article.

## Abbreviations

AOR: Adjusted odds ratio
AIDS: Acquired immunodeficiency syndrome
BMH: Boru Meda Hospital
CI: Confidence interval
CL: Cutaneous leishmaniasis
CNS-IRB: College of Natural and Computational Sciences Institutional Review Board
FNAC: Fine needle aspiration cytology
LCL: Localized cutaneous leishmaniasis
DCL: Diffuse cutaneous leishmaniasis
NTDs: Neglected tropical diseases
OR: Odds ratio
VL: Visceral leishmaniasis
MCL: Mucocutaneous leishmaniasis
PKADL: Post-kala-azar dermal leishmaniasis
SIM: Sudan Interior Mission
SPSS: Statistical Package for Social Sciences
WHO: World Health Organization

## References

[1] Handman E (1999). Cell biology of leishmania. Adv Parasitol..

[2] Claborn DM (2010). The biology and control of leishmaniasis vectors. J Infect Dis..

[3] WHO. Clinical forms of leishmaniasis. Available at: https://www.who.int/leishmaniasis/disease/clinical_forms_leishmaniases/en/index2.html, retrieved 18 Dec 2019.

[4] Marsden PD (1986). Mucosal leishmaniasis (“espundia” Escomel, 1911). Trans R Soc Trop Med Hyg..

[5] Kumari S, Ram VJ (2002). Visceral Leishmaniasis: Clinical features, pathology, diagnosis and chemotherapeutic developments. Drug News Perspect..

[6] WHO. Essential leishmaniasis maps. Available at: [https://www.who.int/leishmaniasis/leishmaniasis_maps/en/], retrieved 27 Nov 2019.

[7] WHO. Leishmaniasis fact sheet. Available at: [https://www.who.int/news-room/fact-sheets/detail/leishmaniasis], retrieved 27 Nov 2019.

[8] CDC. Neglected tropical diseases. Available at: [https://www.cdc.gov/globalhealth/ntd/diseases/index.html], retrieved 27 Nov 2019.

[9] Pires M, Wright B, Kaye PM, da Conceição V, Churchill RC (2019). The impact of leishmaniasis on mental health and psychosocial well-being: a systematic review. PLoS ONE.

[10] Yanik M, Gurel MS, Simsek Z, Kati M (2004). The psychological impact of cutaneous leishmaniasis. Clin Exp Dermatol..

[11] Alvar J, Yactayo S, Bern C (2006). Leishmaniasis and poverty. Trends Parasitol..

[12] Short EE, Caminade C, Thomas BN. Climate change contribution to the emergence or reemergence of parasitic diseases. Infect Dis: Res Treat. 2017;10:1–7. 10.1177/1178633617732296 PMC5755797 29317829

[13] Ponte-Sucre A, Gamarro F, Dujardin J-C, Barrett MP, Lopez-Ve ´lez R, Garcı ´a-Herna ´ndez R, Pountain AW, Mwenechanya R, Papadopoulou B. Drug resistance and treatment failure in leishmaniasis: a 21^st^ century challenge. PLoS Negl Trop Dis. 2017;11(12):e0006052. 10.1371/journal.pntd.0006052 PMC5730103 29240765

[14] Ozaras R, Leblebicioglu H, Sunbul FT, Balkan II, Yemisen M, Sencan I, Ozturk R (2016). The Syrian conflict and infectious diseases. Expert Rev Anti Infect Ther..

[15] Raad II, Chaftari A-M, Dib RW, Graviss EA, Hachem R (2018). Emerging outbreaks associated with conflict and failing healthcare systems in the Middle East. Infect Control Hosp Epidemiol..

[16] Alvar J, Aparicio P, Aseffa A, Den Boer M (2008). Can˜avate C, Dedet J-P, Gradoni L, Horst RT, Lo´pez-Ve´lez R, Moreno J. The relationship between leishmaniasis and AIDS: the second 10 years. Clin Microbiol Rev..

[17] Oumeish OY (1999). Cutaneous leishmaniasis: a historical perspective. Clin Dermatol..

[18] Ashford RW, Bray MA, Hutchinson MP, Bray RS (1973). The epidemiology of cutaneous leishmaniasis in Ethiopia. Trans R Soc Trop Med Hyg..

[19] Lemma A, Foster WA, Gemechu T, Presonton PM, Bryceson A, Minter DM (1969). Studies on leishmaniasis in Ethiopia. I. Preliminary investigations into the epidemiology of cutaneous leishmaniasis in the highlands. Ann Trop Med Parasitol..

[20] Wilkins HA (1972). Studies on leishmaniasis in Ethiopia. VI. Incidence rates of cutaneous leishmaniasis at Meta Abo. Ann Trop Med Parasitol..

[21] Padovese V, Terranova M, Toma L, Barnabas GA, Morrone A (2009). Cutaneous and mucocutaneous leishmaniasis in Tigray, northern Ethiopia: clinical aspects and therapeutic concerns. Trans R Soc Trop Med Hyg..

[22] Morrone A, Pitidis A, Pajno MC, Dassoni F, Latini O, Barnabas GA, Padovese V (2011). Epidemiological and geographical aspects of leishmaniasis in Tigray, northern Ethiopia: a retrospective analysis of medical records, 2005-2008. Trans R Soc Trop Med Hyg..

[23] Bsrat A, Berhe N, Balkew M, Yohannes M, Teklu T, Gadisa E, Medhin G, Abera A (2015). Epidemiological study of cutaneous leishmaniasis in Saesie Tsaeda-emba district, eastern Tigray, northern Ethiopia. Parasit Vector..

[24] Yohannes M, Abebe Z, Boelee E (2019). Prevalence and environmental determinants of cutaneous leishmaniasis in rural communities in Tigray, northern Ethiopia. PLoS Negl Trop Dis..

[25] Tamiru HF, Mashalla YJ, Mohammed R, Tshweneagae GT (2019). Cutaneous leishmaniasis a neglected tropical disease: community knowledge, attitude and practices in an endemic area. Northwest Ethiopia. BMC Infect Dis..

[26] Beyene HB, Alemu M, Degife M (2015). Prevalence and clinical features of cutaneous leishmaniasis in Dembidolo District, Western Ethiopia: a cross-sectional study. Int J Infect Trop Dis..

[27] Negera E, Gadisa E, Yamuah L, Engers H, Hussein J, Kuru T, Hailu A, Gedamu L, Aseffa A (2008). Outbreak of cutaneous leishmaniasis in Silti woreda, Ethiopia: risk factor assessment and causative agent. Trans Roy Soc Trop Med Hyg..

[28] Lindtjorn B (1981). Cutaneous leishmaniasis in the Sidamo highlands. Ethiop Med J..

[29] Mengistu G, Humber DP, Ersumo M, Mamo T (1987). High prevalence of elephantiasis and cutaneous leishmaniasis in Ocholo, south-west Ethiopia. Ethiop Med J..

[30] Mengistu G, Laskay T, Gemetchu T, Humber D, Ersamo M, Evans D, Teferedegn H, Phelouzat MA, Frommel D (1992). Cutaneous leishmaniasis in south-western Ethiopia: Ocholo revisited. Trans R Soc Trop Med Hyg..

[31] Pareyn M, Van den Bosch E, Girma N, van Houtte N, Van Dongen S, van der Auwera G, Massebo F, Shibru S, Leirs H. Ecology and seasonality of sandflies and potential reservoirs of cutaneous leishmaniasis in Ochollo, a hotspot in southern Ethiopia. PLoS Negl Trop Dis. 2019;13(8):e0007667. 10.1371/journal.pntd.0007667 PMC6715250 31425506

[32] Lemma W, Erenso G, Gadisa E, Balkew M, Gebre-Michael T, Hailu A. A zoonotic focus of cutaneous leishmaniasis in Addis Ababa. Ethiopia. Parasit Vector. 2009;2:60. 10.1186/1756-3305-2-60 PMC2794267 19954530

[33] Bekele S, Bekele Y, Mulatu F, Lemma T, Tilahun H, Bizuneh E, Negussie S, Yamuah L, Wassie L, Abebeb M, Aseffa A. Recent trends of cutaneous leishmaniasis in Alert hospital, Addis Ababa. Ethiop Med J Supp1. 2014;37-41. 24696987

[34] Seife T, Benecha AK, Zewdu FT, Ayal A, Misganaw M (2018). Treatment patterns and effectivness of anti-leishmaniasis agents for patients with cutaneous leishmaniasis at Boru Meda Hospital, South Wollo, North East Ethiopia, 2017/18. J Clin Exp Dermatol Res..

[35] van Henten S, Adriaensen W, Fikre H, Akuffo H, Diro E, Hailu A (2018). GV Auwer, van Griensven J. Cutaneous leishmaniasis due to Leishmania aethiopica. E Clinical Medicine..

[36] Hailu A (2006). Di MuccioT, Abebe T, Hunegnaw M, Kager PA. Gramiccia M. Isolation of Leishmania tropica from an Ethiopian cutaneous leishmaniasis patient. Trans Roy Soc Trop Med Hyg..

[37] Assefa A (2018). Leishmaniasis in Ethiopia: a systematic review and meta-analysis of prevalence in animals and humans. Heliyon.

[38] Salam N, Al-Shaqha WM, Azzi A (2014). Leishmaniasis in the Middle East: incidence and epidemiology. PLoS Negl Trop Dis..

[39] Yadon ZE, Rodrigues LC, Davies CR (2013). Indoor and peri-domestic transmission of American cutaneous leishmaniasis in Northwestern Argentina: a retrospective case-control study. Am J Trop Med Hyg.

[40] Oryan A, Akbari M (2016). A review of worldwide risk factors on leishmaniasis. Asian Pacific J Trop Med.

[41] Reithinger RI, Mohsen M (2010). Leslie. Risk factors of anthroponotic cutaneous leishmaniasis at the household level in Kabul, Afghanistan. PLoS Negl Trop Dis..

[42] Lockard RD, Wilson ME, Rodríguez NE. Sex-related differences in immune response and symptomatic manifestations to infection with Leishmania species. J Immunol Res. 2019:4103819. 10.1155/2019/4103819. 10.1155/2019/4103819 PMC6348913 30756088

[43] Ingersoll MA (2017). Sex differences shape the response to infectious diseases. PLoS Pathog..

[44] Travi BL (2002). OsorioY, Melby PC, Chandrasekar B, Arteaga L, Saravia NG. Gender is a major determinant of the clinical evolution and immune response in hamsters infected with *Leishmania* spp. Infect Immun..

[45] Aronson NE, Magill AJ. Leishmaniasis. In: Hunter’s tropical medicine and emerging infectious diseases 10^th^ ed (Ryan ET, Hill DR, ...Endy TP (eds); 2020. 10.1016/B978-0-323-55512-8.00104-6, Pp 776-798, ISBN 978-0-323-55512-8. Elsevier.

[46] Seid A, Gadisa E, Tsegaw T, Abera A, Teshome A, Mulugeta A, Herrero M, Argaw D, Jorge A, Kebede A, Aseffa A (2014). Risk map for cutaneous leishmaniasis in Ethiopia based on environmental factors as revealed by geographical information systems and statistics. Geospatial Health..

[47] Desjeuxe P (2001). The increase in risk factors for leishmaniasis worldwide. Trans Roy Soc Trop Med Hyg..

[48] Stewart CC, Brieger WR (2009). Community views on cutaneous leishmaniasis in Istalif, Afghanistan: implications for treatment and prevention. Int Q Community Health Educ..

[49] Bern CL, Courtenay O, Alvar J (2010). Cattle, sand flies and men: a systemic review of risk factor analysis in South Asia Leishmaniasis and implications for elimination. PLoS Negl Trop Dis..

[50] Dujardin J-C (2000). Risk factors in the spread of leishmaniasis: towards integrated monitoring?. Trends Parasitol..

[51] Votýpka J1, Kasap OE, Volf P, Kodym P, Alten B. Risk factors for cutaneous leishmaniasis in Cukurova region, Turkey. Trans R Soc Trop Med Hyg. 2012;106:186-90. 10.1016/j.trstmh.2011.12.004 22284721

